# Anti-Reflection Nanostructures on Tempered Glass by Dynamic Beam Shaping

**DOI:** 10.3390/mi12030289

**Published:** 2021-03-09

**Authors:** Petr Hauschwitz, Jan Brajer, Danijela Rostohar, Jaromír Kopeček, Tomáš Mocek, Martin Cimrman, Michal Chyla, Martin Smrž

**Affiliations:** 1 HiLASE Centre, Institute of Physics, Czech Academy of Sciences, Za Radnici 828, 25241 Dolni Brezany, Czech Republic; Jan.Brajer@fzu.cz (J.B.); Danijela.Rostohar@fzu.cz (D.R.); Tomas.Mocek@fzu.cz (T.M.); Martin.Cimrman@fzu.cz (M.C.); Michal.Chyla@fzu.cz (M.C.); Martin.Smrz@fzu.cz (M.S.); 2 Faculty of Nuclear Sciences and Physical Engineering, Czech Technical University in Prague, Brehova 7, 11519 Prague, Czech Republic; 3 Institute of Physics of the Czech Academy of Sciences, Na Slovance 2, 18221 Prague, Czech Republic; Jaromir.Kopecek@fzu.cz

**Keywords:** multi-beam micromachning, functionalization, tempered glass, gloss reduction, anti-reflection

## Abstract

Reflectivity and surface topography of tempered glass were modified without any thermal damage to the surroundings by utilizing 1.7 ps ultrashort pulsed laser on its fundamental wavelength of 1030 nm. To speed up the fabrication, a dynamic beam shaping unit combined with a galvanometer scanning head was applied to divide the initial laser beam into a matrix of beamlets with adjustable beamlets number and separation distance. By tuning the laser and processing parameters, reflected intensity can be reduced up to 75% while maintaining 90% of transparency thus showing great potential for display functionalization of mobile phones or laptops.

## 1. Introduction

The value of common everyday products like mobile phones or laptops can be compromised with a poor anti-reflection property of a covering display glass making it unreadable under direct sun. Many methods have been used for the fabrication of antireflection or gloss reduction surface structures including lithography [1], sol-gel [2], etching [3] and multi-stage deposition methods [4]. However, these methods are usually complicated with several steps, long processing times or require chemicals and thus are not environmentally friendly. On the other hand, laser surface micro/nanostructuring offers a flexible, fast and environmentally friendly approach for precise and efficient fabrication of desired micro/nanogeometry in a single step [5].

Laser-made antireflection surface structures are often composed of periodic subwavelength structures [6], laser-induced periodic surface structures (LIPSS) [7] or blind microholes [8] which can alter the refractive index to capture the incoming light. High-resolution of required structures results in longer fabrication times, as well as delicate power-handling close to damage threshold to ensure high-quality processing without thermal damage to the surroundings. To improve the fabrication time and use the laser source more efficiently, multi-beam processing can be applied [9]. However, commonly used diffractive beamsplitters may not provide sufficient freedom in adjusting the spacing between beamlets during process optimization.

In this paper, an ultrashort pulsed laser is used to modify the surface reflectivity by reliable production of sub-wavelength surface structures on tempered glass sample, commonly used as a protective glass layer on displays of mobile phones, smartwatches or laptops. The fabrication speed and process efficiency are improved by a dynamic beam shaping unit combined with a galvanometer scanner allowing to freely divide the beam and adjust a diffraction pattern in real-time. Thus, demonstrating the production of anti-reflection surfaces by dynamic multi-beam glass processing for the first time.

## 2. Materials and Methods

Tempered glass plates with dimensions of 120 × 60 mm and thickness of 500 µm were cleaned in an ultrasonic bath with ethanol before the laser treatment with ultrashort pulsed laser system Perla B (HiLASE Center) emitting 1.7 ps pulses at 1030 nm with the repetition rate of 1 kHz. The output beam was guided into the dynamic beam shaping unit FBS G3 (Pulsar Photonics GmbH) equipped with a spatial light modulator (SLM, Hamamatsu Photonics) and galvanometer scanner (intelliSCAN III 14, Scanlab) with the maximum marking speed of 2 m/s. The beam was focused on a sample with 100 mm telecentric F-theta lens resulting in a spot diameter of 20 µm.

The input beam wavefront was modified by SLM resulting in desired diffractive patterns which are generated in real-time by uploading the pre-calculated computer-generated holograms (phase-masks) on SLM. The final patterns on a sample surface were in a form of orthogonal dot matrixes with the highest possible number of beamlets to fit in the LIDT limit of SLM which is 0.5 mJ for picosecond pulses.

The geometry of fabricated structures was investigated with a scanning electron microscope (SEM) Tescan FERA 3 and laser scanning confocal microscope Olympus OLS5000. Specular reflection and transmission were measured using a spectrometer Green Wave VIS and a tungsten krypton lamp, both coupled into an optical fiber. Precision home-made holders were applied to hold these fibers with respect to the required angles of incidence. The specular reflection and transmission were firstly measured on the plane untreated sample for reference. In the second step, the reflection and transmission were measured on laser patterned samples. By comparing these values, drop in reflection and transmission of the patterned surface is measured in %.

## 3. Results and Discussion

In the first experimental step, the pulse energy and a number of pulses had to be carefully adjusted to selectively modify glass surface in well-defined spot regions without glass cracking and thermal damage. Glass cracking was not observed for pulse energies below 200 µJ (64 J/cm^2^) for a single pulse exposition. However, with a slightly increased pulse count, the glass surface starts to crack (not shown). The optimal pulse energy was determined as 40 µJ (13 J/cm^2^), slightly above the single-pulse damage threshold of 10 J/cm^2^, as the single pulse can still modify the glass surface and no cracks are observed up to 10 consecutive laser pulses.

**Figure 1:**
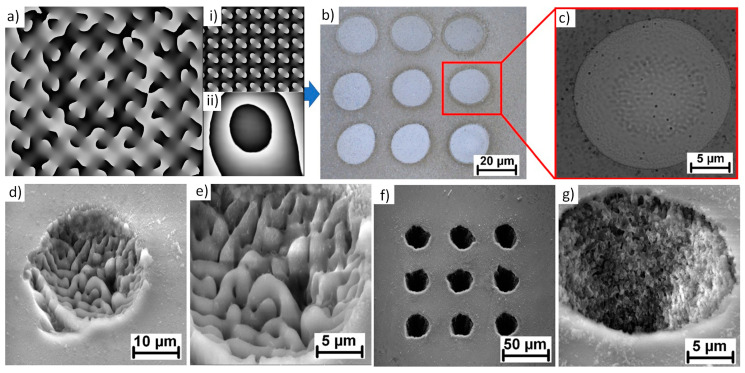
(**a**) Computer generated hologram composed of hologram dividing the initial beam into 3 × 3 matrix (**i**) and correction file compensating SLM flatness (**ii**); (**b**) Final pattern on a glass sample after single pulse exposition with 40 µJ; (**c**) Spot detail indicating sub-wavelength structures in the center part; (**d**) SEM image of a microhole formed after 5 consecutive laser pulses with a pulse energy of 40 µJ, (**e**) detail on a ripple structure inside; (**f**) Microholes drilled with 10 consecutive laser pulses, (**g**) microhole detail with nano-scale features formed on side walls.

Following these observations, the computer-generated hologram responsible for a diffraction pattern dividing the initial beam into the matrix of 3 *×* 3 beamlets was uploaded on the SLM plate (Figure 1a). The pulse energy of the initial beam was then increased up to 420 µJ providing ~40 µJ in each beamlet (85% SLM efficiency). The final pattern of 3 × 3 matrix fabricated on a glass surface in a single pulse is depicted in a microscope image in Figure 1b.

As shown in Figure 1c, an indication of a subwavelength structure was observed in a center of the spot. Therefore, a detail SEM analysis was carried out revealing the ripple structure inside each spot (Figure 1d,e). The lateral spacing of ripple structures is ~900 nm similar to low-frequency laser-induced periodic surface structures [10].

For a single pulse exposition, the ripple structure is formed only in the center part of the spot covering ~60% of the spot diameter. With the increasing number of pulses, ripples are extending to the edges of the spot covering the whole spot in the case of 5 consecutive pulses (Figure 1d,e). With the higher number of pulses, holes can be drilled inside the material (Figure 1f) showing a very high edge quality and nano-scale features on the side walls (Figure 1g).

In the following experimental step, larger areas of 15 × 15 mm were patterned with the optimal pulse energy level of 40 µJ (13 J/cm^2^) and by changing the spot separation distance (HD) in the range of 17 µm to 25 µm and a number of pulses (N) in the range of 1 to 5 pulses. The pitch between beamlets in 3 × 3 matrix was always adjusted to fit with the corresponding HD by uploading a new hologram on SLM.

**Figure 2:**
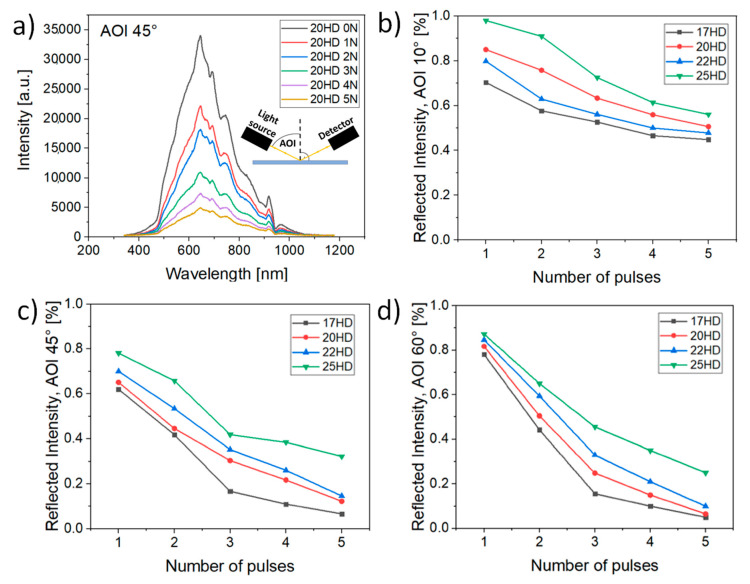
(**a**) Example of reflected intensity curve for an angle of incidence (AOI) = 45° and a different number of applied laser pulses (N); (**b**–**d**) Sample reflection in dependence on the applied number of pulses (N) and different spot separation distance (HD) for the AIO = 10°, 45°, 60°.

As can be observed in Figure 2, different anti-reflection properties can be reached by altering the spot distance and the number of pulses.

This phenomenon might be related to the complex micro and nano topography of the surface which may result in light trapping or diffraction. As it was shown in earlier studies [11], complex micro and nano topography can enhance the absorption of light by multiple reflections and multiple absorptions compared to that of a perfectly flat surface. Similarly, A.Y. Vorobyev et al. [12] observed the decrease in reflection on nanostructured surface features in comparison to that of a polished surface and Korolkov et al. [13] demonstrated the production of antireflective coatings by laser induced nanostructures on metals.

With the increase in the number of pulses, thus with a higher percentage of ripples covering each spot the higher percentage of the incoming light is diffracted or trapped in between surface features. As a result, the reflected intensity can be decreased by more than 50% in the case of AIO = 10° and more than 90% in the case of AIO = 45° and 60°.

**Figure 3:**
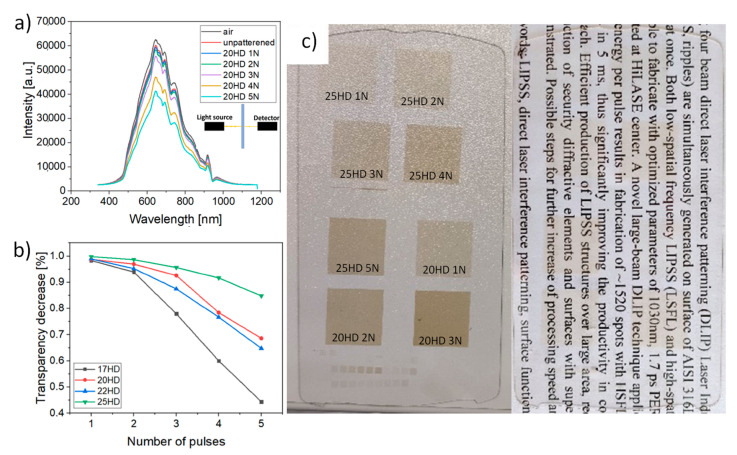
(**a**) Example of transmitted intensity curves for 20HD and different number of applied laser pulses (N); (**b**) Decrease in transparency in dependence on the applied number of pulses (N) and different spot separation distance (HD); (**c**) Photograph of the glass sample on a white table desk and printed paper.

The reflectivity can be also tuned by the spot separation distance. Generally, the closer are the spots, the lower is the reflected intensity as the amount of untreated material decreases. However, the transparency of the structured glass surface decreases as well with the smaller spot distance and the higher number of applied pulses (Figure 3). Therefore, there is a tradeoff between reflectivity and transparency. The detail results of transparency are shown in Figure 3 with a real photograph of structured glass samples.

Following these findings in Figure 2 and Figure 3, the best antireflection results with a high level of transparency above 90%, can be achieved with 20 µm spot separation distance and 3 pulses as it provides the highest drop in reflected intensity, down to 63%, 30% and 25% for AOI of 10°, 45° and 60°, respectively. Thus, showing great potential for display cover glass applications.

## 4. Conclusions

The combination of ultrashort pulse laser and SLM allows fast and flexible fabrication of sub-wavelength ripple structure on a tempered glass surface. The initial laser beam was split into 3 × 3 matrix with adjustable pitch distance increasing the fabrication time nine folds compared to the single beam approach. By adjusting the spot separation distance and number of pulses, different anti-reflection properties can be reached. Up to 75% drop in reflected intensity under AOI = 60° was demonstrated with more than 90% of transparency, thus showing great potential as an alternative to mobile phone or laptop display modification techniques.
